# 25 years of HIV-1 research – progress and perspectives

**DOI:** 10.1186/1741-7015-6-31

**Published:** 2008-10-31

**Authors:** Mark A Wainberg, Kuan-Teh Jeang

**Affiliations:** 1 McGill University AIDS Centre, Jewish General Hospital, Chemin Cote Ste-Catherine, Montreal, Quebec H3T 1E2, Canada; 2 Molecular Virology Section, National Institute of Allergy and Infectious Diseases, National Institutes of Health, Bethesda, Maryland 20892-0460, USA

## Abstract

Twenty-five years after the discovery and isolation of the human immunodeficiency virus by French and American scientists, much progress has been made in basic research, clinical treatment, and public health prevention measures for acquired immunodeficiency syndrome. Here, we summarize, in brief, advances that have been achieved and provide some perspectives on future challenges.

## Background

The first cases of acquired immunodeficiency syndrome (AIDS) were described in homosexual men in the US in 1981 [1]. Several years later in 1983 and 1984, respectively, French and American scientists confirmed that the causative agent for AIDS was a retrovirus, the human immunodeficiency virus (HIV) [2-4]. Today, 25 years after the isolation of HIV-1, approximately 25 million individuals have died from AIDS, a number exceeding by 60 times the total number of American casualties in World War II; and over 33 million people globally are infected and living with HIV-1. In 2007, 2.7 million individuals became newly infected with HIV-1, and 2 million AIDS deaths occurred [5]. Regrettably, half of all people who are infected with HIV acquire the infection before the age of 25 years, and are killed by AIDS before they turn 35. More than 95% of new HIV-1 infections arise in low and middle income nations, populations least likely to have access to antiretroviral therapy. In the face of these daunting statistics and an unabated pandemic, we look back on progress achieved in HIV-1 research, treatment, and policies, and look forward to the challenges that confront AIDS scientists, clinicians and decision-makers for the next 25 years.

### Achievements from basic research

**Figure 1:**
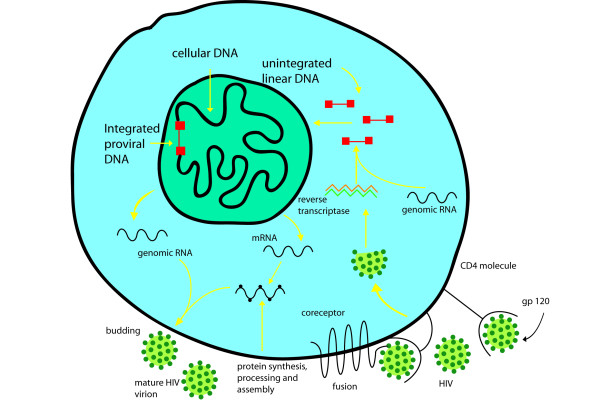
**A schematic illustration of the life-cycle of HIV-1 during the infection of a human cell**. Entry, reverse transcription, nuclear entry, integration, transcription, translation, virus assembly and egress are shown.

The basic research of HIV has made great strides over the past quarter century. Key discoveries, reviewed here in brief, have clarified the intricate steps used by the virus from entry into and exit from the cell (Figure 1). HIV-1 is an enveloped virus with glycoproteins on its surface [6,7], responsible for viral entry into cells. The cell's primary receptor for the virus is the CD4 molecule, and its co-receptors are members of a family of chemokine receptors [8,9]. Using the different co-receptors, HIV-1 can differentially infect T-lymphocytes and/or macrophages [10,11].

Entry of the virus into the cytoplasm initiates the disassembly of the HIV core. At this juncture, the virus can be susceptible to intrinsic cellular antiviral mechanisms which include tripartite motif protein 5a (TRIM5α), a protein with a tripartite motif containing a RING domain, a B-box type 1, and a B-box type 2, followed by a coiled-coil region [12,13], apolipoprotein B mRNA-editing **e**nzyme, catalytic polypeptide-like 3G (APOBEC3G) [14,15], mammalian RNAi (RNA interference) [16,17] and methylation by a cellular methylase enzyme, protein arginine methyltransferase 6 (PRMT6) of the arginine residues [18,19] of several viral proteins. TRIM5α can target the viral capsid protein [13]. APOBEC3G can edit HIV RNA through its cytidine deaminase [20] activity, and in turn HIV encodes an accessory protein called Vif, which counteracts the effect of APOBEC3G [15,21]. Similarly, methylation of viral proteins by cellular methyltransferases like PRMT6 renders them less active and decreases viral replication [22]. Finally, small cellular RNAs appear to contribute to a mammalian cellular defense against retroviral infection [23].

The uncoated HIV-1 reverse transcribes its genomic RNA in the cytoplasm into a DNA copy (a provirus), which is then transported into the nucleus for integration into host chromosomes. Several viral and cellular proteins have been characterized to participate in these processes [24,25]. Once integrated, the provirus is transcribed by the cell's RNA polymerase II machinery with contributions from cellular transcription factors such as Sp1 and NF-κB [26]. Later, viral transcription enters a second stage after the synthesis of the HIV-1 trans-activator of transcription (Tat), an RNA-binding protein. Tat recognizes a viral trans-activation responsive RNA leader [27,28], and together with the cellular positive transcription elongation factor b complex, made up of cyclin-dependant kinase 9 and cyclin T1[29,30], enhances the initiation and processivity of HIV-1 transcription by several hundred fold. Transcribed viral RNAs are next subject to post-transcriptional regulation by HIV-1 Rev and cellular factors Crm1 and RNA helicases [31,32] to export unspliced and partially spliced RNAs from the nucleus into the cytoplasm for translation.

The viral unspliced RNA (around nine kilobases in size) is also the genomic RNA, which is encapsidated into the newly assembled viral particle as a dimer [33,34]. Encapsidation of the dimeric HIV-1 RNA depends on structured RNA motifs [35,36]. The RNA dimer is captured by the viral Gag polyprotein. Thereafter, the assembly and budding of the virus transit via the cell's late endosomal pathway through multi-vesicular bodies [37,38] employing cellular ESCRT (endosomal sorting complex required for transport, proteins that are in multivesicular bodies inside cells) trafficking proteins [39].

### Diagnostics

There has been important progress in regard to HIV diagnostics. From the initial availability of an antibody test, the field has now progressed to be able to monitor directly viral load in the blood using sensitive polymerase chain reaction (PCR) assays. The advent of PCR testing has also represented an important safeguard for the blood supply, since it is now possible to screen all donated blood samples for HIV using this sensitive technique [40].

### Epidemiology

Much has been accomplished also in regard to better understanding the epidemiology of HIV transmission. On a worldwide basis, heterosexual transmission represents the most important means of HIV acquisition [5]. In contrast, however, gay men remain at extremely high risk in regard to new infections in developed country settings. Many observers have questioned whether this may be due to complacency among individuals whose attitude may be that the drugs will work to prolong their lives on an effective basis, regardless of whether or not they eventually do become infected by HIV-1 [5]. It is to be hoped that better awareness and education will provide the basis for diminution of the numbers of new infections, as the realization sets in that drug resistance will likely become a reality over the long term and that the drugs may not continue to work forever. A second consideration is the epidemiologic observation that large numbers of HIV-infected individuals may become prone to a wide array of cancers (for example, lymphomas and anal carcinomas). This is an additional reason for individuals to remain HIV-negative rather than abandoning precautions that would otherwise protect them against HIV-1. The occurrence of high numbers of cancers in HIV-positive individuals is relevant to both developing as well as developed countries.

### Progress in treatment

Without doubt, the most significant headway in the battle against HIV/AIDS has been the development of effective antiretroviral drugs that provide important treatment options for patients infected by HIV. These drugs now exist in a variety of categories based on the enzymatic targets and/or cellular targets that are the targets of the drugs themselves (Table 1). The first drugs were targeted against viral reverse transcriptase but, in short order, we have seen the development of anti-HIV drugs that are targeted against protease, and most recently, integrase inhibitors. The development of combination therapies has been key in assuring prolongation of life, to an extent that HIV disease in almost all developed countries today can be considered to be a chronic manageable condition, unlike the death sentence that existed during the initial years of the epidemic through to the mid 1990s when triple therapies first became available [41,42].

**Table 1:** Antiretroviral drugs approved by the US Food and Drug Administration

**Nucleoside reverse transcriptase inhibitor**	**Non-nucleoside reverse transcriptase inhibitor**	**Protease inhibitor**	**Entry inhibitor**
Zidovudine	Nevirapine	Saquinavir	Maraviroc
Didanosine	Delavirdine	Ritonavir	
Zalcitabine	Efavirenz	Indinavir	**Integrase inhibitor**
Stavudine	Etravirine	Nelfinavir	Raltegravir
Lamivudine		Atazanavir	
Abacavir		Fosamprenavir	**Combinations**
Tenofovir		Tipranavir	Six available, combining two or three drugs
Emtricitabine		Darunavir	
			**Fusion inhibitor**
			Enfuvirtide (T-20)

Entry and fusion inhibitors prevent HIV-1 infection of cells; integrase inhibitor prevents integration of provirus into cellular chromosome.

Of course, it is also fair to state that treatments have improved over the years, not only because of the effectiveness of the drugs themselves but also because newer compounds developed to treat HIV disease are far less toxic than those initially used during the 1980s and 1990s. In addition, simplicity of dosing has become commonplace, such that patients can, in some cases, take only one pill per day, based on a co-formulation that involves two nucleoside/nucleotide reverse transcriptase inhibitors coupled with a non-nucleoside reverse transcriptase inhibitor. The ability to simplify dosing, coupled with current treatment protocols, make current therapies far less toxic than their predecessors, and this has also had important implications in another area, that is, today's treatment regimens are far less prone to the development of drug resistance than were the initially available combination therapies [43].

It will be recalled that the first use of PIs was based on compounds such as saquinavir and indinavir, which at that time were used in their native form. The subsequent development of yet another HIV protease, ritonavir, has been important for the field, since it has been shown that ritonavir can significantly boost plasma levels of other PIs [44]. This has had the consequence of imparting to the newer generation of PIs a much higher genetic barrier for resistance, such that an accumulation of multiple mutations is almost always now required in order for a drug to cease being active against its intended target. This progress has only been possible as a result of continuous research over the past decades that have improved our understanding of drug mechanism of action, and pharmacokinetics and other factors responsible for the development of drug resistance. It is important as well, to recognize the contributions of social science research in this context and the fact that proper adherence to antiretroviral drug regimens has turned out to be a key factor in both the long-term efficacy of the drugs employed in therapy as well as in minimizing the likelihood that drug resistance will develop [45].

### Drug resistance

Drug resistance is probably the single most critical factor responsible for failure of antiretroviral therapy and has ensured that drug companies and scientists are strongly motivated to continue to move forward with new anti-HIV drug discovery programs [46]. In recent years, the search for anti-HIV drugs has been extended to compounds that might act against cellular rather than viral targets. Indeed, one such drug now approved for therapy works by blocking the CCR5 co-receptor that is required for HIV to gain entry into susceptible CD4 lymphocytes [8]. The advantage of a drug that targets cellular proteins rather than viral enzymes is that this should theoretically minimize the likelihood that viral mutagenesis might give rise to drug resistance. Indeed, we now know that other cellular factors might also become important targets for anti-HIV chemotherapy [47]. Good examples of strong leads include the APOBEC3G series of proteins that are impacted by viral proteins such as Vif, and cellular LEDGF proteins that are essential for the proper function of the viral integrase enzyme [15,47].

### Perspectives and challenges

Looking forward, multiple challenges confront HIV-1 researchers, clinicians and policy makers. Below, we highlight a few of these issues.

### Sustainability of antiretroviral treatment

The United Nations estimate that only 7% of people in developing countries have access to antiretroviral drugs, and that only 20% of people worldwide are reached by HIV-1 prevention programs [5]. At recent international conferences on AIDS, the point has repeatedly been made that more people become newly infected by HIV each year than the number that are newly able to gain access to antiretroviral drugs. The drug access that is now being provided by the Global Fund to Fight AIDS, TB, and Malaria and the US-sponsored President's Emergency Plan for AIDS Relief are having major impact in this regard [48]. Nevertheless, it must be recognized that HIV-1 drug treatment is chronic, unabated life-long therapy. Thoughtful policies that save today's lives and countenance the sustained resources needed to continue future long-term treatment have to be developed.

### Prevention strategies, microbicides, and vaccines

Although HIV treatment has improved steadily since the introduction of the first antiretroviral drugs, for example, AZT and ddI, during the late 1980s, most of this progress has been limited to developed countries. In contrast, access to anti-HIV drugs continues to be problematic in most of the developing world. Sadly, for millions of HIV-infected individuals, access to anti-HIV therapy is not yet a reality [5]. Anti-HIV drugs may contribute much to the prevention of infection. As an example, it is well-known that the most important correlate of HIV transmission is the level of plasma viremia within an infected individual. Since anti-HIV drugs act to limit viral replication, they also lower plasma viremia, and it is hoped to levels that are non-detectable by very sensitive PCR assays [49]. As a consequence, patients whose plasma viremia is well suppressed can potentially be regarded as non-infectious for sexual contacts. This statement, although somewhat controversial in terms of public health recommendations, establishes rationale to treat patients on as widespread a basis as possible, in order to lower viral loads and hopefully diminish overall levels of HIV transmission. This is important, since practical progress in regard to prevention strategies involving HIV-1 has been almost negligible [50].

In regard to preventive HIV vaccines, two phase 3 clinical trials, one involving the viral protein gp160, and a second, involving a recombinant adenovirus vector containing viral proteins, have failed [51]. Indeed, the latter trial may have resulted in a greater number of individuals in the experimental rather than in the placebo arm becoming infected. There is widespread consensus that it will be at least another decade before phase 3 trials are ready to begin once again with vaccines that are designed to prevent new HIV transmissions.

Similarly, there has been frustration in regard to the use of anti-HIV vaginal microbicides to prevent infection. A large number of phase 3 clinical trials, involving non-specific polyanionic substances, have failed [50]. These have included trials of nonoyxynol-9, sulfated polysaccharides, and other substances; in which again, it appears that the women who received the anti-HIV microbicides may actually have suffered greater numbers of infections than women in the control arm of the studies, perhaps because the substances used were toxic to vaginal mucosal tissue, thus promoting infection in the experimental arm.

It is now unlikely that further studies using non-specific approaches of the sort mentioned above will be undertaken. Rather, there is strong consensus that future microbicide trials should be conducted with antiretroviral drugs that could perhaps be formulated as vaginal gels and that might protect against HIV transmission from an infected male partner. A type of compound that probably has the strongest rationale in this context is a CCR5 antagonist; several such compounds have now been shown in animal models to be protective against transmission of simian immunodeficiency virus. Whether or not CCR5 antagonists formulated as gels will also protect against HIV transmission is not yet known. In addition, there is also rationale to develop certain nucleoside(tide) and/or non-nucleoside reverse transcriptase inhibitors as anti-HIV vaginal microbicides. Research in this area is continuing apace to evaluate these various prevention strategies [52].

An approach similar to the above, also now undergoing phase 3 clinical testing, is that of pre-exposure prophylaxis (PREP) [53]. People who are susceptible to HIV infection are asked to take antiretroviral drugs on a prophylactic rather than therapeutic basis. Clinical studies now underway are evaluating the co-formulation of tenofovir together with FTC (Truvada). The reason for choosing these two drugs in combination is that both of them have relatively long half-lives, meaning that dosing is only required once per day. The hope is that these drugs will be able to interfere with early reverse transcription events that may transpire immediately after infection, thereby preventing the virus from being able to take root within the body. Results of these studies should be available within 1 to 2 years.

One issue that must be considered, however, is drug resistance. Of course, it may be difficult to assess whether or not antiretroviral drugs when used as preventives will work, if the viruses being transmitted by infected partners are themselves drug resistant. Also, there is a danger that an individual may choose to avail themselves of this PREP approach while being in the window period for seroconversion, at a time that high viral loads are present, thus representing a scenario whereby selection of drug resistance might occur. This notwithstanding, many observers now consider the PREP approach to be the most promising of all strategies for prevention of HIV transmission [52].

### Pediatric infections

Almost all HIV-infected pregnant women in developed countries now receive triple therapy during the course of pregnancy to prevent mother-to-child transmission of the virus. This has resulted in the virtual elimination of the pandemic of native-born pediatric AIDS in all developed country settings. Sadly, such progress has not been realized in developing countries [5].

### Circumcision

Another success story in relation to prevention has been the understanding that male circumcision may help to reduce female-to-male transmission of HIV by as much as 60% [54]. This is presumably because circumcision eliminates the foreskin that contains penile tissue rich in Langerhans cells that are thought to be important portals of entry for HIV into the body. Hence, elimination of this tissue may diminish the likelihood of transmission.

## Conclusion

The theme of World AIDS Day in 2007 was 'leadership'. As AIDS moves from the 20th into the 21st century, the world today with its increased globalization is much different than it was in 1983; and over the next 25 years, it will change again. For example, several well-regarded economic sources have projected that China will have overtaken the US by 2040 to become the world's largest economy, with India capturing third place. Although China's official AIDS statistics in 2005 reveal a relatively few, 650,000, HIV-cases, against a total population of 1.3 billion people, China's future HIV numbers will undoubtedly increase significantly [55]. It seems inescapable that a long-term solution for HIV/AIDS must include a commitment of leadership and resources from China, on a scale no weaker than the recent support of the Olympic Games.

Our achievements over the past 25 years, as outlined above, have come, in part, because of outstanding, capable leaders in all facets of HIV-1 research. A concern discussed at the International AIDS Society's XVII International AIDS Conference in Mexico City was how to develop, nurture, and avoid a shortfall in new AIDS leaders. Realistically, major initiatives such as the development of successful prophylactic HIV-vaccines and effective topical antiviral microbicides require perseverance that spans a decade or more with little reason to expect short-term success. Thus, looking ahead, we must attain sustainability of economic resources in order to assure delivery of antiretroviral treatment in developing countries, while not neglecting the need to develop the new human leadership that will be essential to sustain and accelerate the global campaign against HIV/AIDS.

## Abbreviations

AIDS: acquired immunodeficiency syndrome; APOBEC3G: apolipoprotein B mRNA-editing **e**nzyme: catalytic polypeptide-like 3G; ESCRT: endosomal sorting complex required for transport; HIV: human immunodeficiency virus; PCR: polymerase chain reaction; PI: protease inhibitor; PREP: pre-exposure prophylaxis; PRMT6: protein arginine methyltransferase 6; Tat: trans-activator of transcription; TRIM5α: tripartite motif protein 5a.

## Competing interests

The authors declare that they have no competing interests.       

## Authors' contributions

Both MAW and KTJ contributed equally to the writing of this commentary.

## Pre-publication history

The pre-publication history for this paper can be accessed here:


